# How to differentiate between Eosinophilic Granulomatous Polyangiitis and Henoch-Schönlein Purpura

**DOI:** 10.31138/mjr.33.1.88

**Published:** 2022-03-31

**Authors:** Dinesh Abhijeeth Shanker, Clive Kelly, Anupam Paul, Sara Elhardari

**Affiliations:** 1 Northern General Hospital, Sheffield Teaching Hospitals NHS Foundation Trust,; 2 James Cook University Hospital, Middlesbrough, Cleveland, UK

**Keywords:** ANCA vasculitis, small vessel vasculitis, asthma, rash

## Abstract

We present a 29-year-old gentleman admitted with eight years of recurrent, bilateral, erythematous, macular rash involving the legs, occurring every summer. Other symptoms included intermittent joint aches with dysaesthesia. He was investigated previously with a renal biopsy, a skin biopsy and endoscopy and had been diagnosed as Henoch-Schönlein Purpura (HSP). However, his past medical history included progressive asthma and sinusitis. Investigations revealed peripheral eosinophilia, positive rheumatoid factor and elevated IgE levels with mild renal impairment. During admission, he became hypoxic. A CT pulmonary angiogram (CTPA) showed changes consistent with early pulmonary manifestations of EGPA, which improved on a repeat scan after commencing the patient on high dose prednisolone. Revising the diagnosis, his condition fulfilled criteria for ANCA negative EGPA rather than HSP.

## INTRODUCTION

Eosinophilic Granulomatosis with Polyangiitis (EGPA), formerly known as Churg– Strauss syndrome, is an autoimmune, anti-neutro-phil cytoplasmic antibody (ANCA)-associated vasculitis with an incidence of 0.6–6.8 cases/million and an incidence of approximately 35–64 cases/10^6^ person-years in asthmatics.^1,2^ The Revised International Chapel Hill consensus conference nomenclature of vasculitides held in 2012 defined it as an eosinophil-rich and necrotising granulomatous inflammation, often involving the respiratory tract, and necrotising vasculitis predominantly affecting small- to medium-sized vessels, associated with asthma and eosinophilia.^3^ The disease has been described to have three phases: the allergic phase, the eosinophilic phase, and the vasculitic phase. These phases need not proceed in succession, and a patient may not present with all the three phases. Phase one is characterised by asthma and or allergic rhinitis with or without nasal polyps, which precedes full blown disease by many years. In the second phase, eosinophilic infiltration of various tissues, including gastrointestinal tract, myocardium, and upper and lower respiratory tracts occur. Systemic vasculitis sets in during the final phase of the disease, marked by necrotising small vessel vasculitis.^4^

## CASE REPORT

**Figure 1.**
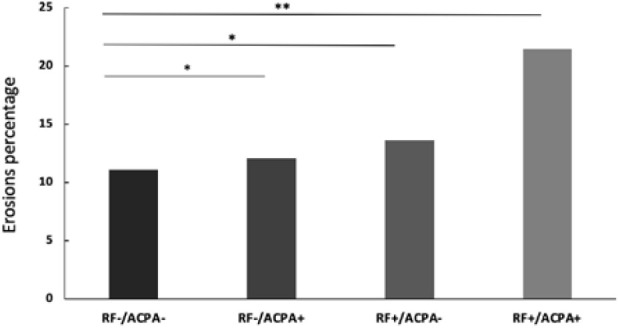
Non-blanching, non-palpable erythematous macular rash on admission.

A 29-year-old gentleman, with a long history of asthma, was admitted with a recurrent rash (**Figure 1**) on his legs extending from his ankle to mid-thigh which had been present for one week. He was unable to bear weight and complained of painful dysaesthesia and sensory loss. He has had multiple episodes every summer since 2012 and had had several hospital admissions. On admission he complained of pain in the ankles, and described intermittent early morning stiffness affecting his elbows, knees, feet, and ankle joints for years.

Past medical history included bronchial asthma since childhood which is well controlled on inhalers with no asthma-related hospital admissions. He is a non-smoker and drinks alcohol occasionally. No relevant family history. He denied allergies and regular medications include Fostair 100/6 one puff twice daily added 4 weeks prior to admission with spacer.

On examination, a non-blanching, non-palpable erythematous macular rash was present bilaterally over the anterior aspect of the legs and extended up to the aspect of the thigh with synovitis of the ankles. Lower limb power and sensation were preserved. Chest examination revealed wheeze with a few fine basal crepitations, uninfluenced by coughing.

His notes revealed that he had been diagnosed as having Henoch-Schönlein Purpura (HSP) on previous admissions. He had a skin biopsy reported as leucocytoclastic vasculitis without any IgA deposition. He had undergone a renal biopsy in 2014 when he presented with haematuria with deteriorating renal function and suspected Immunoglobulin A (IgA) nephropathy. However, light and electron microscopy showed no abnormalities with negative immunofluorescence. He had also been investigated for coeliac disease with duodenal biopsy, which was reported as showing nonspecific intraepithelial lymphocytes without villous atrophy. He also described features of intermittent sinusitis.

On admission he had a normal white cell count of 9.8 cells/mm^3^ with an eosinophilia of 3.1%. He had previously had a peripheral eosinophilia of 7.8%. Renal function tests indicated stage 2 CKD (eGFR – 79ml/min/1.73), with a creatinine of 126umol/l. Vasculitis screen including ANCA and MPO was negative with modestly elevated Ig M levels at 2.34 g/l but normal IgA and complement levels. Rheumatoid factor was elevated (793 IU/ml) with negative CCP antibodies. Urinalysis showed a trace of proteinuria. Three days after admission, the patient had a single episode of haemoptysis and was noted to be hypoxic (pO_2_ = 8.2 kPa).

We checked D-dimer which was modestly elevated (0.49). We proceeded to CTPA to exclude pulmonary embolism (PE). This showed no evidence of PE but did demonstrate patchy centrilobular ground-glass opacification with upper zone predominance. COVID PCR test was negative and a High-Resolution CT (HRCT) confirmed findings suggestive of Eosinophilic Granulomatosis with Polyangiitis (EGPA) with focal peribronchovascular consolidation. Immunoglobulin E levels were very elevated at 195 KU/l. An echocardiogram showed normal left ventricular size and function with mild concentric hypertrophy.

**Figure 2.**
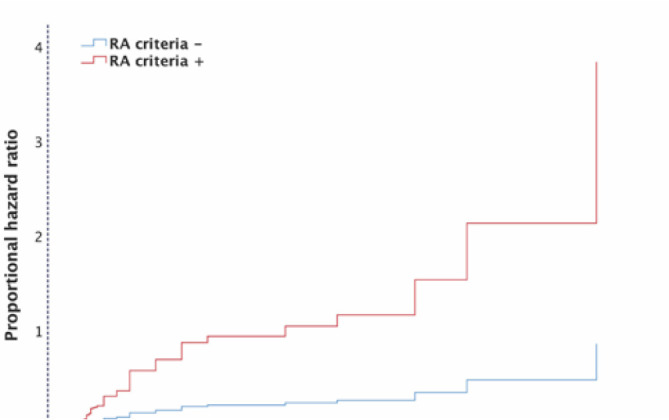
CT Pulmonary Angiogram showing patchy centrilobular ground-glass opacification with upper zone predominance.

Based on the American College of Rheumatology (ACR) criteria for EGPA,^4^ of peripheral eosinophilia, asthma, pulmonary infiltrates, paranasal abnormalities, neuropathy, and extravascular eosinophilia on biopsy, we suspected this diagnosis rather than HSP. He was referred for review of the skin biopsy for eosinophilia as other criteria were met, together with his elevated IgE. With reference to the previous suspicion of HSP, his rash was non-palpable and non-purpuric. Taken together with normal IgA, negative skin, renal and duodenal biopsies for IgA deposition, he did not meet any criteria for this diagnosis.

The patient was started on prednisolone at 60mg which was tapered over 8 weeks, together with Mycopheno-late mofetil at a dose of 500mg BD was increased up to 2g after 8 weeks. All his symptoms resolved, and he remained well at follow-up with normal FBC and IgE levels. His rash, chest, and neurological features remained resolved. His Five factor score of 0 suggested that he didn’t meet the criteria for cyclophosphamide.^1,5^

In patients with EGPA, pANCA is positive in 40–60% of cases. EGPA can be divided into clinically relevant ANCA negative and ANCA positive phenotypes. Patients who are ANCA positive on diagnosis tend to have symptoms involving the kidneys, skin, and peripheral nerves (mononeuritis multiplex), while ANCA negative patients had higher incidence of endocardial and myocardial involvement on follow-up and higher incidence of lung infiltrates. ANCA negative patients were noted to have higher levels of eosinophilia and more severe attacks of asthma. It seems likely that in ANCA positive patients, the dominant mechanism of the pathogenesis is antibody mediated while in the ANCA negative disease manifestation may be due to eosinophilic infiltration.^1^ EPGA were found to have elevated IgE levels especially in the vasculitic phase of the disease and elevated rheumatoid factor likely due to the autoimmune process.^6^

Glucocorticoids play a key role in the treatment of EGPA. Patients with life- and/or organ-threatening disease manifestations, need to be given other agents such as cyclophosphamide along with glucocorticoids. Azathioprine and methotrexate maybe used as agents for maintaining remission.^5^ Mycophenolate mofetil has also shown to be successful in treating EPGA along with glucocorticoids.^7^ Omalizumab, an anti-human IgE monoclonal antibody, has proven to be beneficial in treating patients with moderate to severe asthma and helps to taper down steroids.^2^

## DISCUSSION

The Five-Factor score (FFS) is a French scoring devised to look at the prognosis of the patient. It has 5 parameters, of which any of the following (age >65 years, cardiac involvement, gastrointestinal haemorrhage, or peak creatinine of 150 μmol/L), is associated with poor prognosis, while the 5^th^ factor (sinusitis) is associated with a good prognosis. Patients with any adverse feature may require adjunctive cytotoxic drugs.^5^

HSP is an IgA mediated autoimmune vasculitic disorder affecting small vessels with multisystem involvement with an annual incidence ranging from 6.3 to 70.3 per 100,000. It usually affects children but may also occur in adults. The classic tetrad of symptoms in HSP includes a palpable purpuric rash in the legs and buttocks, arthralgia/arthritis, renal and gastrointestinal symptoms. HSP is a self-limiting condition in the majority of patients and requires symptomatic management. In severe cases, patients are given steroids (oral or IV) and immunosuppressive drugs, such as Cyclophosphamide and Azathioprine, which are added only in rare cases of rapidly progressive glomerulonephritis or pulmonary haemorrhage.^8–10^ It is most common during winter^9^ and IgA plays a cardinal role in its pathogenesis. IgA deposits may be found on the vessel walls causing leucoclastic vasculitis and renal mesangium, leading to IgA nephropathy.^10^

Our patient had the clinical and investigative features of an IgE mediated vasculitis (EGPA) rather than an IgA mediated disorder (HSP).

## ABBREVIATIONS

ANCA: Anti-neutrophil cytoplasmic antibody
CTPA: CT pulmonary angiogram
EGPA: Eosinophilic Granulomatous Polyangiitis
HSP: Henoch-Schönlein Purpura
Ig: Immunoglobulin
